# Global Citizens – Global Jet Setters? The Relation Between Global Identity, Sufficiency Orientation, Travelling, and a Socio-Ecological Transformation of the Mobility System

**DOI:** 10.3389/fpsyg.2021.622842

**Published:** 2021-03-30

**Authors:** Laura S. Loy, Josephine Tröger, Paula Prior, Gerhard Reese

**Affiliations:** ^1^Department of Social, Environmental and Economic Psychology, Faculty of Psychology, University of Koblenz-Landau, Landau, Germany; ^2^Institute of Psychology, University of Hamburg, Hamburg, Germany

**Keywords:** global identity, sufficiency orientation, travelling, pro-environmental behaviour, policy support, mobility, socio-ecological transformation, flight shame

## Abstract

Global crises such as the climate crisis require fast concerted action, but individual and structural barriers prevent a socio-ecological transformation in crucial areas such as the mobility sector. An identification with people all over the world (i.e., *global identity*) and an openness toward less consumption (i.e., *sufficiency orientation*) may represent psychological drivers of a socio-ecological transformation. We examined the compatibility of both concepts as well as their relation to people’s support of a decarbonised mobility system and their flight mobility behaviour – a CO_2_-intensive behaviour that may be particularly difficult to refrain from for globally identified people, but less so for sufficiency-oriented people. In an online study conducted in Germany (*N* = 317), we found that global identity and sufficiency orientation were positively related. Both were negatively related to past flight-related CO_2_ emissions and positively related to refraining from flying and the support of decarbonised mobility policies. Accounting for both showed that sufficiency orientation in particular was related to fewer flight-related CO_2_ emissions and refraining from flying. Furthermore, we examined people’s travel experiences. While global identity was unrelated to the frequency and duration of international travelling, it was positively related to the frequency and quality of contact with local people met on journeys. An experimental variation of whether participants first answered questions on global identity or on travel experiences revealed that remembering past international travelling led to higher reported levels of global identity. Taken together, global identity seems to profit from in-depth international contact with people, but can be decoupled from resource-intensive travel behaviour. Globally identified and sufficiency-oriented people may support a socio-ecological transformation. Our results indicate a compatibility of global identity and sufficiency orientation. Experimental and longitudinal research should examine causal links to foster our understanding of the conditions under which both can be strengthened.

## Introduction

Global crises such as climate change are challenging humanity as a whole and collective efforts from people all over the world are required to build a sustainable future. A sustainable future, however, seems at odds with the current status of the planet. Global environmental change has reached levels that surpass a safe operating space for humanity (Rockström et al., 2009; Steffen et al., 2015; O’Neill et al., 2018). It is evident that together with technological developments, a socio-structural transformation is necessary (Abson et al., 2017; Fischer and Riechers, 2019). Paths include less resource-intensive behaviour patterns, particularly in affluent countries, but also political measures that remove structural constraints and provide structural incentives for such behavioural changes. Our psychological perspective addresses potential drivers of transformation on the level of behavioural *niches* (Geels, 2004). Specifically, we focus on the domain of (air) mobility and potential psychological predictors of individual and system change.

Previous research suggests that an identification with all humanity as an inclusive ingroup (i.e., *global identity*; McFarland et al., 2019) might motivate people to engage for a socio-ecological transformation (e.g., Reese, 2016). Global identity is related to people’s engagement for a socio-ecological transformation in the form of pro-environmental behaviours and policy support in various studies (e.g., Renger and Reese, 2017; Brieger, 2019; Joanes, 2019; Loy and Reese, 2019), but less is known about how people develop a global identity (see McFarland et al., 2019, for an overview). One possibility that has been discussed is travelling and meeting people from all over the world (Sparkman and Eidelman, 2018; Römpke et al., 2019). However, air travelling allowing such contact is amongst the most CO_2_-intensive and unsustainable individual behaviours. At the same time, it is strongly embedded within the current socio-technical system: flying is comparably cheap, readily available, and often faster than other means of transport.

**FIGURE 1:**
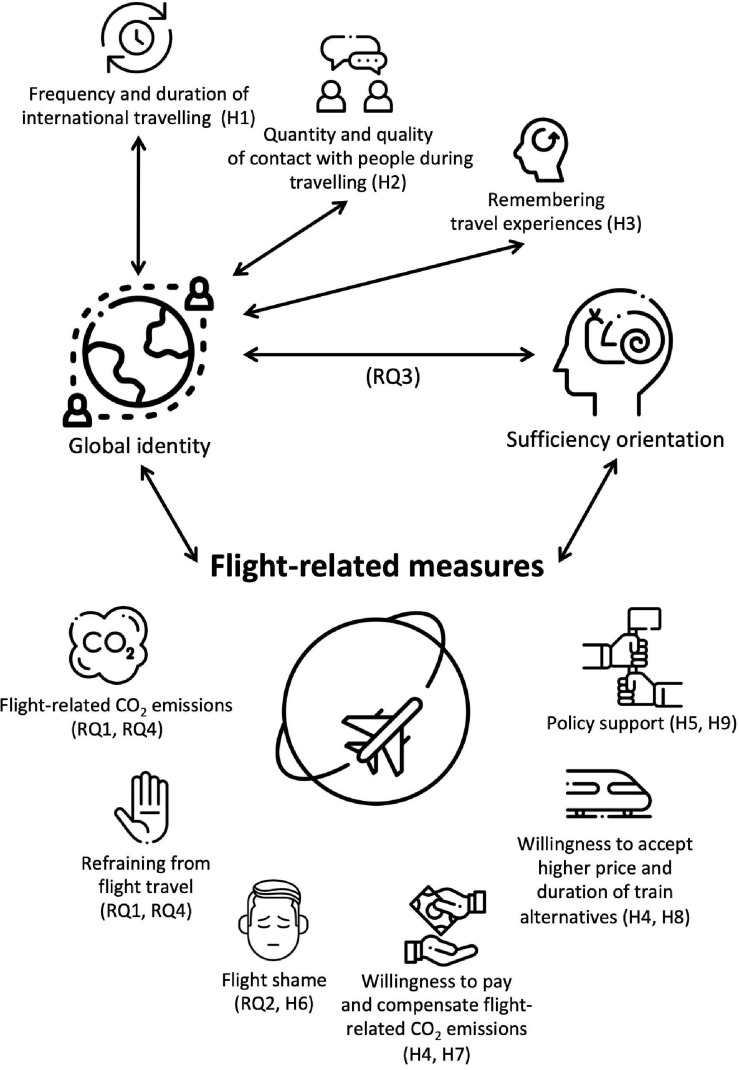
Graphical overview of research questions and hypotheses. RQ, research question; H, hypothesis. This graphical illustration has been designed using resources from Flaticon.com. Icons are by Freepik (www.freepik.com), Dan Darius (www.flaticon.com/authors/darius-dan), Pixel perfect (www.flaticon.com/authors/pixel-perfect), and Pixelmeetup (www.flaticon.com/authors/pixelmeetup).

The overarching goal of our research is thus to investigate the relation between global identity, travel behaviour and experiences, as well as the support of political measures that transform and decarbonise the mobility system. In addition, we test whether global identity is compatible with *sufficiency orientation* (i.e., the attitudinal stance to refrain from consumption; Verfuerth et al., 2019), and whether one or the other is more strongly related to people’s willingness to refrain from flying and to support a socio-ecological transformation of the mobility system. Figure 1 provides a graphical overview of our research.

## Theoretical Background

### Travelling in the Current Mobility System

Mobility is a human need, but within our (affluent western) society, being on the move is often coupled with climate-damaging CO_2_ emissions. In 2010, transportation caused an estimated 14% of the global greenhouse gas emissions (IPCC, 2015). Air travelling produces far more emissions compared to other forms of mobility. For instance, one air trip from Berlin to Paris causes approximately 260 kg CO_2_ equivalents; taking the train would produce only 40 kg (KlimAktiv, 2020). In 2019, international aviation contributed 2.4% to global greenhouse gas emissions (Crippa et al., 2019). Moreover, recent research suggests that aviation’s contribution to atmospheric warming is even larger, namely “three times the rate of that associated with aviation CO_2_ emissions alone when calculated as net effective radiative forcing” (Lee et al., 2021, p. 2). These emissions, however, seem to be caused by a relatively small share of the most frequent travellers who have the means to fly (i.e., money, social status, see e.g., Gössling et al., 2017). Hence, if the majority of humankind flew, this would increase flight emissions drastically: Predictions for the year 2050 suggest that commercial aircraft emissions might triple (EESI, 2019) and account for a quarter of the global carbon budget (Graver et al., 2019). A decarbonisation of the mobility system and a change in the way we are travelling is essential in order to limit climate change (Urry, 2008; European Commission, 2011; Zipori and Cohen, 2015). Given the current technological infrastructure, people can deliberately reduce their mobility-related CO_2_ footprint by simply travelling less and/or by choosing less CO_2_-intensive means of transport such as trains. Moreover, they can support policy measures that make CO_2_-intensive travel options comparably less attractive (e.g., carbon pricing, investment in public transport network; Maestre-Andrés et al., 2019).

Many people are aware of the climate crisis and express willingness to contribute to climate change mitigation (UBA, 2019; European Commission, 2020). Two thirds of the European population state that they are ready for a shift to more environmentally friendly modes of transport (e.g., public transport; European Commission, 2020). However, these intentions often do not translate into actual behaviour change (Lassen, 2010; Alcock et al., 2017; Geiger et al., 2018). One reason for this might be that infrastructural and political incentives are promoting non-ecological choices: Flying is judged as much faster, more convenient, and less expensive compared to alternative options (European Commission, 2020). Flight travelling has become an essential part of the western globalised culture (Castillo-Manzano and López-Valpuesta, 2014; McDonald et al., 2015). Moreover, global interconnectedness and long distance travelling are perceived requirements in many professions, although they are not necessarily related to professional success (e.g., in academia, Wynes et al., 2019). At the same time, travelling with resource-intensive means is increasingly seen as contradictory to ecological values within our society and calls for a socio-ecological transformation of the mobility system become louder (Gössling et al., 2020).

Understanding how this mobility system may transform requires a perspective that accounts for the different layers of a complex system. According to the multilevel perspective outlined by Geels (2004), a system that determines societal functioning comprises three levels. The level of the *regime* consists of current institutions (e.g., governmental agencies), infrastructures (e.g., airports and public transport system), technologies (e.g., drive technologies), and policies (e.g., regulations regarding carbon pricing), but also normative behavioural practices (e.g., frequent flying). The regime is embedded in the *landscape*, which consists of “the technical, physical and material backdrop that sustains society” (Geels and Schot, 2007, p. 403), such as the climatic conditions or the availability of fossil resources. While regime and landscape are seen as rather stable, new technologies, behavioural practices, and ideas for policy change can evolve on the level of *niches*. Here, networks of individuals emerge, who promote societal change through changing their own behaviour or through supporting political change. Our research is situated on this level of niches. We examine psychological predictors of people’s mobility behaviour and their support of policy measures toward a socio-ecological transformation of the mobility system. Specifically, we investigate the role of global identity and sufficiency orientation as drivers for transition processes.

### Global Identity and Travel Experiences

Different conceptualisations of a *global identity* exist (see McFarland et al., 2019; Carmona et al., 2020, for an overview). In our research, we refer to the concept labelled identification with all humanity, introduced by McFarland et al. (2012) and further differentiated by Reese et al. (2015, see also Reysen and Hackett, 2016; Hamer et al., 2020). It comprises a *global self-definition* (i.e., a definition of oneself as part of a community consisting of people all over the world) and a *global self-investment* (i.e., a concern for and solidarity with people all over the world). The concept is rooted in social identity theory (SIT, Tajfel and Turner, 1979), which states that a substantial part of who we are is defined by our group memberships. We identify with our so-called ingroups and differentiate ourselves from outgroups. Self-categorisation theory (SCT, Turner et al., 1987) further assumes that we can define our identity on three levels, namely personal identity, social group identity, and – on the highest level – human identity. Identifying on this highest level goes along with perceiving oneself as part of an ingroup encompassing all humanity. A further theoretical basis comprises theories of personal growth, which assume that caring for all humans characterises a mature person (Adler, 1927/1954; Maslow, 1954; see McFarland et al., 2012, 2013; Reese et al., 2015, for an in-depth discussion). Identities can be understood as traits we develop over time. Hence, individuals differ in how strongly they identify with all humanity (McFarland et al., 2012). However, resonating with SIT/SCT, different parts of our identity, including our global identity, can be more or less salient in a context and guide our perceptions and actions (Turner et al., 1987; Reese et al., 2015; McFarland et al., 2019; Loy and Spence, 2020; Sparkman and Hamer, 2020).

Past research has discussed how a global identity could emerge (see McFarland et al., 2019, for an overview). One plausible reasoning based on intergroup contact theory (Pettigrew and Tropp, 2006; Pettigrew et al., 2011) was that personal contact with people all over the world might strengthen global identification (see e.g., Sparkman and Eidelman, 2018; Römpke et al., 2019). Supporting this rationale, Römpke et al. (2019, Study 1) found that German participants who had come into (fictitious) contact with a person from another continent through a simulated Internet chat program reported higher levels of global identity compared to a control group. Moreover, the amount of international contacts students reported in a questionnaire predicted their global identity in a follow-up assessment 6 months later Römpke et al. (2019, Study 2). Sparkman and Eidelman (2018, Study 2) found that what they labelled as “contact with cultural members” was positively related to United States citizens’ global identity. Sparkman and Hamer (2020) found positive correlations of a similar composite measure with global identity in a Polish sample. None of these studies particularly addressed travel experiences abroad. In our research, we aimed to extend prior findings in this regard and predicted:

H1: The more international travel experiences people have made (frequency and duration of staying abroad), the stronger their global identity.

In another study, Sparkman and Eidelman (2018, Study 3) asked United States participants about the “quantity and quality of one’s intercultural contact” (see also Römpke et al., 2019). Both aspects were positively related to global identity. We transferred this idea to experiences with local people met during travelling and predicted:

H2: The higher the contact quantity (H2a) and quality (H2b) with local people during travelling, the stronger people’s global identity.

Beyond examining correlations between global identity and travel experiences, we aimed to gain causal insights. SCT (Turner et al., 1987) supposes that a global identity may be triggered by cues that evoke associations with it (McFarland et al., 2019; Loy and Spence, 2020). We reasoned that thinking about past travel experiences might be such a cue and experimentally varied whether participants in our study first answered questions on travel experiences or on global identity, respectively. Even though this cannot give firm causal evidence that travelling impacts global identity, it could be a first hint that (remembering) respective experiences make(s) global identity more salient. We predicted:

H3: Remembering travel experiences raises the salience of global identity.

### Global Identity and Decarbonised Travelling

Past research has reasoned that a global identity might be related to people’s motivation to address global environmental crises (e.g., Batalha and Reynolds, 2012; Reese, 2016). Positive relations were found with pro-environmental attitudes (e.g., Reysen and Katzarska-Miller, 2013; Lee et al., 2015; Reysen and Hackett, 2016; Assis et al., 2017), pro-environmental behavioural intentions and behaviours (e.g., Der-Karabetian et al., 2014; Lee et al., 2015; Rosenmann et al., 2016; Renger and Reese, 2017; Joanes, 2019; Leung and Koh, 2019; Loy and Reese, 2019), and the support of pro-environmental policies and organisations (e.g., Leung et al., 2015; Brieger, 2019; Loy and Reese, 2019).

Some of these previous studies included items on mobility behaviour that were, however, only investigated as part of an overall lifestyle. Alcock et al. (2017) reported results of a United Kingdom survey study, in which pro-environmental attitudes were related to household behaviours but not to people’s non-work-related flights (see also Lassen, 2010; McDonald et al., 2015). Hence, flight-reduction might constitute a particularly difficult behaviour regardless or despite of its high CO_2_-saving potential – especially for people highly identified on a global level. Travelling to distant locations might be particularly attractive for them so that they rather focus on other pro-environmental behaviours (e.g., a plant-based diet) to express their motivation to address climate change. Accordingly, Römpke et al. (2019, Study 2) found that global identity was positively related to the intention to avoid animal products but not air travel. In other words, the empirical evidence on a relation between global identity and pro-environmental outcomes might lead to the supposition that flight reduction is also a likely goal pursued by globally identified people. However, their global orientation might conflict with this goal. In line with the latter supposition, Oswald and Ernst (2020) found that a cosmopolitan identity (i.e., a multidimensional concept including one dimension similar to our global identity conceptualisation) was positively related to flight kilometres in the last year. Due to little empirical evidence and opposing plausible theoretical rationales, we examined the relationship between global identity and flight behaviour in terms of past flight-related CO_2_ emissions and how often people refrained from flying:

RQ1: Is global identity related to past flight-related CO_2_ emissions (RQ1a) and refraining from flight travel (RQ1b)?

Recent media coverage on the Fridays for Future movement coined the term *flight shame* in order to grasp people’s reaction to protesters’ frequent appeal that global jet setting is one of the most CO_2_-intensive behaviours and should be reduced (Gössling et al., 2020). Moral emotions such as shame and guilt have been found to be related with pro-environmental behavioural intentions and behaviours (Mallett, 2012; Harth et al., 2013; Rees et al., 2015). We therefore additionally assessed flight shame and asked:

RQ2: Is global identity related to flight shame?

Beyond flying behaviour, we also examined how willing people were to compensate flight-related CO_2_ emissions (i.e., carbon offsetting) and switch to alternative train options. As these behaviours do not oppose long-distance travelling *per se*, we expected, in line with the results on a relation between global identity and pro-environmental behaviours cited above:

H4: The stronger people’s global identity, the higher their willingness to compensate flight-related CO_2_ emissions (H4a) at higher costs (H4b), pay more for alternative train options (H4c) and accept longer travel durations of alternative train options (H4d).

Finally, we aimed to go beyond individual behaviour and examined people’s support of a socio-ecological transformation of the mobility system. Based on prior research that found a positive relation between global identity and climate policy support including mobility-related changes (Loy and Reese, 2019), we predicted:

H5: The stronger people’s global identity, the stronger their support of policy measures that decarbonise the mobility system.

As outlined above, global identity could conflict with the willingness to fly less despite a principal willingness to reduce one’s CO_2_ impact. One might hope that more resource-efficient technologies will solve this conflict in the future (e.g., through electrification). However, it has become evident that technological progress alone cannot reduce carbon emissions from travelling to a satisfactory extent (Peeters and Dubois, 2010) and fundamental behaviour shifts are necessary. Therefore, the concept of sufficiency addresses the idea of absolute consumption reduction. In the following, we argue that individuals’ sufficiency orientation might (additionally or even better) explain why people refrain from flying.

### Sufficiency Orientation, Global Identity, and Decarbonised Travelling

Sufficiency is an increasingly discussed concept in several disciplines (Gorge et al., 2015; Spangenberg and Lorek, 2019; Toulouse et al., 2019; Tröger and Reese, 2021). Introduced as one essential part of the sustainability strategy bundle comprising efficiency, consistency, and sufficiency, it encompasses the shrinkage of absolute resource consumption levels (Darby and Fawcett, 2018; Linz, 2004). Understanding the development and role of an attitudinal stance, namely people’s so-called *sufficiency orientation*, may be a prerequisite for consumption change (Spangenberg and Lorek, 2019; Verfuerth et al., 2019). Only a few studies examined sufficiency orientation as predictor for actual behaviour (Verfuerth et al., 2019) and we know little about commonalities and differences to other concepts that predict pro-environmental behaviour.

Theoretically, sufficiency orientation and global identity might be positively related because they share strong social justice motives (see Howell, 2013; Schäpke and Rauschmeyer, 2014; McFarland et al., 2019). Both are related to pro-environmental attitudes and behaviours (e.g., Loy and Reese, 2019; Verfuerth et al., 2019). The specific case of flight behaviour, however, might reveal a difference and possible incompatibility of these two concepts. As outlined above, global identity is positively related to pro-environmental behaviour in general, but evidence with regard to flying is unclear. Globally identified people may experience a conflict between an interest to travel and the environmental damage this might cause if fossil-based travel modes are used. Sufficiency-oriented people, on the contrary, may experience such conflicts to a lesser extent. As their attitudinal stance is strongly rooted in consumption reduction, their priority might lie on refraining from behaviour that has a high ecological impact. Due to these contradicting theoretical arguments, we explored:

RQ3: Is sufficiency orientation related to global identity?

A study by Moser and Kleinhückelkotten (2018) showed that pro-environmental identity (i.e., the self-description as a resource-saving person) positively correlated with so-called intent-oriented behaviour (i.e., self-reported estimations of personal efforts to save natural resources) but not with impact-oriented behaviour (e.g., frequency of long-distance vacations). We argue that a stronger sufficiency orientation should be related with refraining from flying because it consists of the conviction that less overall consumption is necessary to protect the climate and the environment. Qualitative research showed that people who are sufficiency-oriented in fact use fewer resources in their everyday routines (Speck and Hasselkuss, 2015). A more recent study showed that the stronger people’s sufficiency orientation, the lower their carbon impact regarding food consumption, electricity consumption, and everyday mobility, while air travelling was unrelated (Verfuerth et al., 2019). Due to the fact that empirical results have so far failed to confirm the theoretical predictions, we asked:

RQ4: Is sufficiency orientation related to past flight-related CO_2_ emissions (RQ4a) and refraining from flight travel (RQ4b)?

The discussion around sufficiency is conceptually grounded in justice theory and in practical sustainability science (see Spengler, 2016, for an overview). The idea is to define and meet minimum and maximum thresholds of consumption that enable a fair and just distribution of resources now and in the future in accordance with the earth’s natural limits (Syme and Nancarrow, 2012; Schäpke and Rauschmeyer, 2014; Alexander, 2019). While only few people, mainly from affluent societies, have the means to fly, environmental consequences mostly affect people not responsible for the emissions (e.g., O’Neill et al., 2018). People who are sensitive to this injustice experience moral emotions such as guilt and shame (e.g., Schmitt et al., 2010). Therefore, we predicted:

H6: The stronger people’s sufficiency orientation, the more flight shame they experience.

As argued above, sufficiency-oriented people may not feel the need to travel by airplanes and therefore also no need to compensate flights in terms of carbon offsetting. Furthermore, compensation policies have been criticised as a strategy to morally licence environmentally harmful behaviour that could involve backfiring effects (i.e., flying even more; Font Vivanco et al., 2018; Sorrell et al., 2020). This should be at odds with the moral standards of sufficiency-oriented people. Instead, they might support resource-saving alternatives to flight travel. We thus predicted:

H7: The stronger people’s sufficiency orientation, the lower their willingness to compensate flight-related CO_2_ emissions.

H8: The stronger people’s sufficiency orientation, the higher their willingness to pay more for alternative train options (H8a) and accept longer travel durations of alternative train options (H8b).

Finally, sufficiency as a sustainability strategy calls for adequate policy instruments to cut back emissions through infrastructural change (Toulouse et al., 2019; Tröger and Reese, 2021). Prior research found a positive relation between sufficiency orientation and policy support in the field of plastic consumption (e.g., taxation of plastic, Heidbreder et al., unpublished data). As sufficiency-oriented people may feel particularly responsible for their own consumption and perceive a corresponding agency (Speck and Hasselkuss, 2015), they may critically reflect on current structural constraints that hinder low-carbon individual behaviour. Therefore, we assumed that they support structural policy measures allowing people to better enact their sufficiency-oriented intentions and predicted:

H9: The stronger people’s sufficiency orientation, the stronger their support of policy measures that decarbonise the mobility system.

## Materials and Methods

### Design and Procedure

We followed the APA guidelines for the ethical conduct of research. Participants answered an online questionnaire programmed with SoSci Survey (Leiner, 2019). Inclusion criterion was that they lived in Germany for at least 5 years. We raffled four 25€ vouchers as incentive. After giving informed consent, participants were randomly assigned to one of two experimental groups. They either first answered questions on global identity (see section “Global Identity”, *control condition*) or on travel experiences (see section “Travel Experiences,” *salience condition*). Then, they answered all other questions, followed by a debriefing.

### Participants

We conducted an *a priori* power analysis (see Supplementary Section “Power Analysis”) and recruited a convenience sample of *N* = 322 participants (see Supplementary Section “Participant Characteristics” for socio-demographic details) through snowball sampling via personal contacts of several student assistants, mostly via Facebook and WhatsApp. We also used university Facebook groups and Facebook groups focusing on empirical research participation. Excluding *n* = 5 participants (see Supplementary Section “Exclusion of Outliers and Implausible Values”) resulted in a final sample of *N* = 317 used for our analyses (257 females, 58 males, 2 diverse; *M* = 28.4 years of age, *SD* = 10.0, range = 18–65). On a 5-point scale assessing the subjective income situation (Buerke, 2016), only few stated limited resources by indicating 1 (*not enough by half*, *n* = 4) or 2 (*just make ends meet*, *n* = 25). The majority evaluated their financial situation as satisfactory, indicating 3 (*overall doing well*, *n* = 121), 4 (*well looked after and can afford quite a lot*, *n* = 141), or 5 (*do not have to restrict myself in any way*, *n* = 26). We also assessed monthly household income, but could not use this variable due to a programming mistake in the online questionnaire.

### Measures

In the following, we provide an overview on the self-report measures used to answer our research questions (see Supplementary Section “Measures” for detailed descriptions and Supplementary Tables 1, 2 for psychometric properties)^1^. It took participants on average 18.5 min to fill out the questionnaire. All variables are provided on the OSF Forum^2^, the key scales in Supplementary Section “Measures.”

#### Global Identity

We used an adapted version (see Loy and Reese, 2019 and Supplementary Section “Global Identity”) of the Identification with all Humanity Scale (IWAH, McFarland et al., 2012; Reese et al., 2015). Participants stated their agreement with five statements, respectively, on global self-definition and global self-investment on a 7-point scale.

#### Travel Experiences

We asked participants how often in the past 5 years they had travelled in Europe on average per year on a 7-point scale, how long their respective longest stay had been, how often in their lives they had travelled outside of Europe on a 7-point scale, and again, how long their respective longest stay had been (Supplementary Section “Travel Experiences,” see Sparkman and Eidelman, 2018, for a similar measure). We used a measure by Islam and Hewstone (1993) to assess the quantity and quality of contact with people met during travelling on 7-point scales with five items, respectively (Supplementary Section “Travel Experiences,” see also Sparkman and Eidelman, 2018).

#### Flight-Related Measures

##### Flight-related CO_2_ emissions

First, people indicated if they had travelled by airplane at least once in the last 5 years. Those who had flown (*n* = 291) next indicated if they had travelled more than five times per year. We categorised those travelling less than five times as *occasional flyers* (*n* = 219) and asked them to list all their flights in the last 5 years into a provided entry mask (i.e., departure location and destination). We categorised those travelling more than five times per year as *frequent flyers* (*n* = 72) and asked them to estimate their average number of flights per year for seven distance categories. We provided reference destinations for each category. Based on this information, we calculated the individual CO_2_ emissions (in tons per person) using an online footprint calculator (see Supplementary Section “Calculation of Flight-Related CO_2_ Emissions”). The values of *n* = 15 cases were incomplete and we excluded them from further analyses (see Supplementary Section “Exclusion of Outliers and Implausible Values”).

##### Refraining from flight travel

We asked participants how often in the past 5 years they had refrained from flying on a 7-point scale and what their motives were (see Supplementary Section “Refraining From Flight Travel”).

##### Flight shame

Participants indicated their agreement to the statements “I feel ashamed/guilty that I have travelled by airplane” on 7-point scales (see Supplementary Section “Flight Shame”). The *n* = 26 participants who had not flown did not receive this question (missing values).

##### Willingness to pay and compensate flight-related CO_2_ emissions

We asked participants to imagine that they travel by plane and pay 100€. They indicated whether they would pay a CO_2_ compensation in terms of carbon offsetting on a 7-point scale (*not in any case* to *in any case*) and how much money they would pay on a visual analogue scale (*0€* to *100€*). We excluded *n* = 4 cases (missing values).

##### Willingness to accept higher price and duration of train alternatives

We confronted participants with the scenario to travel within Europe, deciding whether to use the train as alternative to a 2h flight costing 100€. They indicated the maximum amount of money they would pay for the train (in €) and the maximum duration they would accept (in hours). We excluded the values of *n* = 6 cases (2 missing values, 4 outliers; see Supplementary Section “Exclusion of Outliers and Implausible Values”).

#### Policy Support

We refined and extended a policy support scale used by Loy and Reese (2019, see also Tobler et al., 2012, Supplementary Section “Policy Support”) to focus only on mobility-related measures. On a 7-point scale, participants rated five restrictive measures relating to cars, three restrictive measures relating to flying, and three supportive measures relating to public transport and train travelling.

#### Sufficiency Orientation

We measured sufficiency orientation with six items from the sufficiency orientation short scale, capturing people’s attitude toward a low-carbon lifestyle (Henn, 2015; Verfuerth et al., 2019) and added six items capturing people’s conviction that consumption reduction is a necessary means to environmental and climate protection. Participants stated their agreement on a 7-point scale (see Supplementary Section “Sufficiency Orientation”).

## Results

The results regarding our research questions (RQ) and hypotheses (H) in terms of bivariate correlations are summarised in Table 1 (see Supplementary Table 2 for all bivariate correlations).

**TABLE 1:** Bivariate correlations addressed in our research questions and hypotheses.

**Variable**	**RQ/H global identity**	**1**	**2**	**RQ/H sufficiency orientation**	**3**	**4**
**Global identity**						
1. Global self-definition^a^						
2. Global self-investment^a^		0.94*				
**Sufficiency orientation**						
3. Low-carbon lifestyle^a^	RQ3	0.44*	0.47*			
4. Consumption impact^a^	RQ3	0.42*	0.49*		0.80*	
**Travel experiences**						
5. Frequency of travelling Europe^b^	H1	0.03	0.03			
6. Duration of travelling Europe	H1	–0.05	–0.05			
7. Frequency of travelling beyond Europe^b^	H1	0.08	0.07			
8. Duration of travelling beyond Europe	H1	0.10	0.10			
9. Quantity of contact with locals^a^	H2a	0.24*	0.21*			
10. Quality of contact with locals^a^	H2b	0.27*	0.27*			
**Decarbonised mobility practices and appraisals**						
11. Flight-related CO_2_ emissions	RQ1a	–0.08	−0.12*	RQ4a	−0.14*	−0.15*
12. Refraining from flight travel	RQ1b	0.22*	0.25*	RQ4b	0.39*	0.31*
13. Flight shame	RQ2	0.35*	0.40*	H6	0.46*	0.45*
14. Willingness CO_2_ compensation	H4a	0.34*	0.39*	H7	0.39*	0.36*
15. Amount CO_2_ compensation	H4b	0.21*	0.22*	H7	0.20*	0.17*
16. Accepted train price	H4c	0.15*	0.16*	H8a	0.22*	0.19*
17. Accepted train travel duration	H4d	0.13*	0.12*	H8b	0.17*	0.17*
18. Policy support^a^	H5	0.43*	0.48*	H9	0.65*	0.65*

*p < 0.05. We used pairwise exclusion of missing cases. ^*a*^Factor scores resulting from CFA were used. ^*b*^Spearman correlations were calculated for these ordinal variables, all others are Pearson correlations.

### Global Identity and Travelling

Disconfirming H1, frequency and duration of past international travelling outside of Germany in Europe and beyond were not related to either global identity dimension. However, confirming H2, the quantity and experienced quality of contact with local people met on journeys were positively related to both global self-definition and global self-investment. A regression analysis with all travel measures as parallel predictors of global identity (overall mean score), controlling for gender, age, and subjective income situation, confirmed the small relations of contact quantity and quality with global identity (see Table 2).

**TABLE 2:** Results of regressing global identity (mean score) on travel experiences.

	***B***	***SE***	***p***	**95% CI**	**β**	***R*^2^**
						0.135
Constant	4.45	0.55	<0.001	[3.23, 5.60]		
Gender	–0.41	0.18	0.020	[−0.79, −0.04]	−0.13*	
Age	–0.01	0.01	0.198	[−0.02, 0.00]	–0.07	
Subjective financial situation	–0.11	0.08	0.171	[−0.28, 0.05]	–0.07	
Frequency of travelling Europe	0.00	0.04	0.911	[−0.07, 0.08]	0.01	
Duration of travelling Europe	–0.00	0.00	0.134	[−0.00, 0.00]	–0.09	
Frequency of travelling beyond Europe	–0.01	0.03	0.782	[−0.07, 0.06]	–0.02	
Duration of travelling beyond Europe	0.00	0.00	0.206	[−0.00, 0.01]	0.08	
Quantity of contact with locals^a^	0.11	0.05	0.030	[0.01, 0.24]	0.14*	
Quality of contact with locals^a^	0.28	0.08	<0.001	[0.09, 0.46]	0.21*	

*p < 0.05. Confidence intervals (CI) were bootstrapped through 5,000 samples. Gender was dichotomised as 1 (*female*) and 2 (*male*); *n* = 2 participants indicating *diverse* were omitted in these analyses due to the low case number. ^*a*^Mean scores were used.

Comparing people who had answered the questions on travel experiences before and after answering questions on global identity revealed that thinking about past travelling led to higher reported levels of global self-definition (global identity salience condition: *M* = 5.26, *SD* = 1.27; control condition: *M* = 4.87, *SD* = 1.32; *t*(315) = 2.68, *d* = 0.30, *p* = 0.008), but not to statistically significant higher levels of self-investment (*d* = 0.15, *p* = 0.170). Even though the effect size was small, this indicates that (remembering) international experiences might raise the salience of a global ingroup and partly confirms H3.

### Global Identity and Decarbonised Travelling

Global self-investment but not self-definition was negatively related to past CO_2_ emissions resulting from flying (RQ1a). The stronger people’s global self-investment and self-definition, the more they had refrained from flying (RQ1b), the more flight shame they experienced (RQ2), the more they were willing to compensate flight-related CO_2_ emissions (confirming H4a) at higher costs (confirming H4b), and to accept higher prices (confirming H4c) and durations of alternative train options (confirming H4d). The relations were small to medium. Moreover, they more strongly supported policy measures for a mobility system that restricts flying and car use and promotes public transport (confirming H5, medium to strong relations).

### Global Identity, Sufficiency Orientation, and Decarbonised Travelling

Global identity was positively related to sufficiency orientation (RQ3, medium to strong relations). Sufficiency orientation showed a similar pattern of small to medium correlations to mobility-related measures: It was negatively related to flight-related CO_2_ emissions (RQ4a) and positively related to refraining from flying (RQ4b), flight shame (confirming H6), acceptance of higher train travel durations (confirming H8a) and prices (confirming H8b), and the support of mobility-related policy measures (confirming H9; strong relations). Disconfirming H7, sufficiency orientation was also positively related to the willingness to compensate flight-related CO_2_ emissions at higher costs.

We additionally ran regression models with global identity and sufficiency orientation as parallel predictors of past flight-related CO_2_ emissions, willingness to reduce flying, and policy support favouring a transformed mobility system to examine their relative explanatory value (see Table 3). We used mean scores because the dimensions were highly correlated, and regarding them as separate predictors would have posed the problem of collinearity. Moreover, we controlled for gender, age, and subjective income situation. These analyses showed that, when accounting for both constructs, only sufficiency orientation predicted fewer CO_2_ emissions and the willingness to refrain from flying. Both global identity and sufficiency orientation predicted policy support^3^.

**TABLE 3:** Results of regressing the flight-related measures and policy support on global identity and sufficiency orientation (mean scores).

	***B***	***SE***	***p***	**95% CI**	**β**	***R*^2^**
	Flight-related CO_2_ emissions					0.032
Constant	52.00	26.08	0.047	[−2.38, 186.81]		
Gender	–0.86	7.69	0.911	[−21.54, 15.77]	–0.01	
Age	0.09	0.30	0.768	[−0.39, 0.80]	0.02	
Subjective financial situation	4.93	3.62	0.175	[0.75, 10.33]	0.08	
Global identity	–0.83	2.94	0.777	[−18.31, 5.89]	–0.02	
Sufficiency orientation	–8.34	3.87	0.032	[−19.53, 0.39]	−0.15*	
	Refraining from flight travel					0.164
Constant	–1.87	0.87	0.032	[−3.32, −0.28]		
Gender	0.25	0.26	0.337	[−0.30, 0.78]	0.05	
Age	0.01	0.01	0.538	[−0.02, 0.03]	0.03	
Subjective financial situation	0.10	0.12	0.405	[−0.16, 0.35]	0.04	
Global identity	0.13	0.10	0.182	[−0.08, 0.33]	0.08	
Sufficiency orientation	0.76	0.13	<0.001	[0.50, 0.98]	0.36*	
	Policy support					0.475
Constant	0.31	0.41	0.455	[−0.50, 1.19]		
Gender	–0.17	0.12	0.171	[−0.45, 0.10]	–0.06	
Age	–0.00	0.00	0.700	[−0.01, 0.01]	–0.02	
Subjective financial situation	0.07	0.06	0.199	[−0.04, 0.19]	0.05	
Global identity	0.16	0.05	<0.001	[0.07, 0.26]	0.17*	
Sufficiency orientation	0.71	0.06	<0.001	[0.59, 0.83]	0.57*	

*p < 0.05. Confidence intervals (CI) were bootstrapped through 5,000 samples. Gender was dichotomised as 1 (*female*) and 2 (*male*); *n* = 2 participants indicating *diverse* were omitted in these analyses due to the low case number.

## Discussion

### Summary of the Results and Theoretical Contribution

Our research investigated the relation between global identity, travelling, and the support of a decarbonised mobility system. In our German sample, frequency and duration of travelling outside of Germany was not related to global identity. However, frequency and quality of contact with local people met on journeys correlated positively with both global identity dimensions. Global self-investment but not self-definition was negatively related to flight-related CO_2_ emissions. The stronger people’s global self-definition and self-investment, the more they had refrained from flying and the more they supported policy measures that restrict flying and car use and promote public transport.

Moreover, we examined whether global identity is compatible with sufficiency orientation and found positive relations of both global identity dimensions with people’s attitude favouring a low-carbon lifestyle and their conviction that consumption reduction is a necessary means to environmental and climate protection. Sufficiency orientation showed a similar pattern of correlations with flight-related outcomes. Accounting for both constructs showed that sufficiency orientation in particular predicted lower flight-related CO_2_ emissions and refraining from flying. It more strongly predicted policy support.

In sum, global identity seems to profit from in-depth international contact with people, but can be decoupled from resource-intensive travel behaviour. It appears to be compatible with the willingness to consume less and with supporting political measures toward a decarbonised mobility system. However, sufficiency orientation was the statistically stronger predictor. We therefore suggest that global identity could be promoted in combination with sufficiency orientation in order to gain support for a socio-ecological transformation of the mobility system.

Our study provides three major contributions to the research field. First, it shows that a positive contact with local people during journeys is related to global identity, rather than frequent travelling. Second, it brings together research on two evolving concepts within environmental psychology that share strong relations with pro-environmental action and shows that they are compatible: global identity and sufficiency orientation. Third, it suggests a new approach to increase global identity salience in a particular situation. We experimentally varied whether participants first answered questions on global identity or on personal travel experiences. Thinking about past travelling led to higher reported levels of global self-definition. Hence, (remembering) international experiences might raise the salience of a global ingroup, contributing to the few published studies that successfully raised global identity salience (Reese et al., 2015, Study 3; Römpke et al., 2019).

### Limitations and Future Research Directions

First, given our correlational design, we cannot draw causal conclusions whether the quantity and quality of contact with locals strengthen global identity, whether the direction is vice versa, or caused by unconsidered third variables. Experimental research involving contact situations suggests that international contact can increase global identity (Römpke et al., 2019). However, it could also reasonably be argued that globally identified people seek and are more receptive to positive international contact. Longitudinal studies assessing political ideologies (e.g., right-wing authoritarianism) suggest bi-directional relations between such constructs and presumably dependent variables (Onraet et al., 2014). Similarly, we cannot infer causality in the relations between global identity, sufficiency orientation, and mobility behaviours and policy support. Experimental or longitudinal approaches may shed light on their mutual effects.

Second, our convenience sample was very young, mostly female, highly educated, and subjectively in a satisfactory financial situation. We suspect that the awareness regarding aviation’s contribution to climate change is comparably high within this group of people and that our results should not yet be generalised. Future studies should replicate our findings within more heterogeneous and, optimally, randomly selected representative samples. We also suggest to include measures of both objective and subjective income situations. It is still an open question to which extent sufficiency orientation is related to or developed independently from people’s economic status. Likewise, global identity, the willingness to pay for carbon offsetting or costly train options, and the support of certain policy measures such as taxes might depend on people’s financial situation.

Third, our research involved self-report measures. Even though a recent study showed that social desirability biases do not seem to be huge in studies on pro-environmental behaviour (Vilar et al., 2020), observational measures could complement our approach in follow-up studies (Lange and Dewitte, 2019).

Related to this point, it is possible that memory retrieval of participants’ flights caused some distortions in the CO_2_ emission calculations. We decided to consider a period of 5 years in order to not only cover recent lifestyles (which might have changed, e.g., due to child birth), but a more representative picture. For frequent flyers, we asked for the average number of flights per year for seven distance categories instead of listing all flights separately in order to avoid frustration and drop-outs due to memory difficulties. Future studies could try to use trace data or GPS data from airlines (Graver et al., 2019). Still, we believe that our study provides a more precise measurement approach than prior studies, which often assessed self-reported frequencies of flights only (e.g., “Over the last 12 months, how many times did you travel by plane for personal reasons?”, Schubert et al., 2020).

Our experimental variation of question order (global identity measured after vs. before remembering international experiences) raised the salience of a global ingroup. Communication research could build on this finding and examine how to evoke travel memories. If this strengthens global identity, it might encourage recipients’ collective engagement for a socio-ecological transformation.

### Practical Implications

#### Cultivating and Communicating Global Identity and Sufficiency Orientation

Our correlational results suggest that people with a strong global identity have not been abroad more often – and even fly less – than people with a lower global identity. Thus, global identity does not seem to contradict a low-carbon lifestyle. One might further ask how a global identity could be fostered in accordance with decarbonised travelling? We suggest that the focus should lie on creating opportunities that allow people from different parts of the world to meet and engage in meaningful contact.

Exchange programmes (e.g., the European Erasmus programme) can provide opportunities to establish in-depth contacts with locals through living in a foreign country. We suppose that study or working stays can bring rewarding contact with locals for both sides. Organisations that fund such stays could structurally support ecological travel modes (i.e., encourage and fund train arrival). However, it has to be kept in mind that these opportunities are not equally available to everyone as they depend on unequally distributed financial and social resources (Urry, 2012; Schubert et al., 2020). Therefore, access should be promoted for people of more diverse social backgrounds from all over the world.

In addition, extending international platforms via the Internet may provide contact opportunities even in remoter areas (Amichai-Hamburger and McKenna, 2006). Hence, “digital pen friendships” might be a further pathway to develop a global identity (Römpke et al., 2019). Moreover, playing characters and thereby virtually experiencing the lives of people all over the globe in a virtual simulation game fostered global empathy (Bachen et al., 2012). We imagine that such a game could also cultivate global identity. Finally, recent research suggests that mind-body practices (i.e., yoga, meditation) might foster global identity, because it is a goal of these techniques to strengthen the perceived connectedness of all living beings, even without meeting them in person (Brito-Pons et al., 2018; Loy and Reese, 2019).

Our findings further suggest that sufficiency orientation and global identity do not contradict each other. People holding these orientations not only share the motivation to protect the environment but also share a common lifestyle, in our case the preference for low-carbon travelling. Therefore, we suggest that both orientations could be cultivated and communicated at the same time. Practitioners could think about how global identity could be made salient through communicative means (see e.g., Loy and Spence, 2020). Our results suggest that making people think about past travel experiences might be one way to do so. Hence, writers and journalists could try to evoke such memories with their narratives. Moreover, they could add images or information about the idea of consuming less. An applied example is the online initiative “terran”^4^. The campaign creates vivid images of low-carbon travelling through stories, pictures, and funny sayings from people all over the world. It could thus make global identity salient, while exemplifying ways of travelling in the spirit of sufficiency orientation.

Finally, our results indicate that sufficiency orientation in particular is linked to a strong desire for structural change through policy measures. It is thus possible that strengthening sufficiency orientation in our society would accelerate a socio-ecological transition. This could be achieved by arguing against the negative connotation of renunciation and the potential fear of “the less” through emphasising social and ecological benefits (Tröger and Reese, 2021). Recent evidence suggests that norms toward flying already shifted in the German society due to the global Fridays for Future movement and the European-wide flight shame debate (Koos and Naumann, 2019; Gössling et al., 2020). This might explain why we found a relation between sufficiency orientation and reduced air travelling unlike Verfuerth et al. (2019), who conducted their study before these movements. This social norm shift might help to promote a sufficiency orientation in the future. Sufficiency is not a lifestyle that expresses itself through seclusion or solitude, but rather through the desire to contribute to climate protection by reducing consumption and living a frugal life within a connected and globalised world. The idea of “less is more” can be used in campaigns that promote decarbonised forms of travelling.

#### Toward a Sustainable Mobility System

Referring back to the multi-level model of Geels (2004), changes in the *landscape*, such as the planetary boundaries we are approaching or have already surpassed (Steffen et al., 2015), call for a system transformation to ensure a good life for all in the future. The decarbonisation of the mobility system is one goal to reach this vision (European Commission, 2011). Policy changes on the level of the *regime* (Geels, 2004) can promote changes in individual behaviour (e.g., reduced car or aviation use). These policy measures could consist of taxes (e.g., taxation of gasoline-based cars or kerosene), banning of technologies (e.g., abolition of combustion engine), or removing subsidies (e.g., reduced value added tax to fuel oil; see Kanger et al., 2020). Moreover, policy measures can establish decarbonised infrastructures and change the socio-technical system. For example, a case study in Lisbon showed that simply expanding and completing the cycling network in the city centre and the introduction of an electric bike-sharing system lead to a large increase of cyclists (Félix et al., 2020). An expansion of cycling routes is now attempted in many metropolitan areas (e.g., Paris, Berlin, and Bógota). Similarly, the (re)introduction of attractive (night) train connections could help to replace flight travel (Baumeister and Leung, 2020; for a respective initiative, see “Back on Track”)^5^.

Engagement on the level of *niches* (Geels, 2004) seems important to generate innovative ideas and to establish bottom-up acceptance for policy measures. Kanger et al. (2020) thus suggest to stimulate and accelerate niches, for example, through research and development funding schemes, creating innovation platforms, or market-based policy instruments. In line with this suggestion, online portals for citizen participation, in which people are asked to share their ideas for a future mobility system, or workshops in which citizens are actively involved in the development of mobility concepts could guide a transition process (e.g., Gebhardt et al., 2019). Moreover, apps for car and bike sharing (Cellina et al., 2019) or the free availability of cargo bikes (Becker and Rudolf, 2018) could be useful instruments to engage people in using alternative low-carbon modes for mobility. For non-urban areas, however, these niches require political support: While there certainly is a vast amount of mobility infrastructure available, it is often limited to promoting individual car mobility. Infrastructures allowing communal transportation, especially in terms of car and bike sharing, but also increased public transport would require public support schemes, both for users and providers of such options alike.

We argue that beyond these measures to stimulate niches from the “outside,” it is a key to understand people (in those niches and beyond) as essential part of the socio-technical system and ask: What motivates them to support a system change? Which psychological prerequisites does a change need? Our research shows that global identity and sufficiency orientation are psychological correlates of people’s support of a decarbonised mobility system in terms of concrete actions and the support of structural changes.

### Conclusion

Our study suggests that a global identity benefits from international contact and is nevertheless compatible with the willingness to consume less, including carbon-intensive forms of travelling. Given the extent and drastic development of the climate crisis, CO_2_ emissions from travelling need to be reduced and decarbonised alternative travel models should be promoted in the future (e.g., slow travel, Dickinson et al., 2011). Global identity and sufficiency orientation seem to be compatible with these goals. Although our correlational data cannot claim causality, we still cautiously suggest that cultivating these orientations might be paths toward a society that practices more sustainable forms of mobility. How they evolve and how they can stimulate each other are questions for future research.

## Data Availability Statement

The datasets presented in this study can be found in online repositories. The names of the repository/repositories and accession number(s) can be found below: OSF Forum (https://bit.ly/3vbEGvh).

## Ethics Statement

Ethical review and approval was not required for the study on human participants in accordance with the local legislation and institutional requirements. The patients/participants provided their written informed consent to participate in this study.

## Author Contributions

LL, JT, PP, and GR developed the idea, theoretical background, and research design. PP programmed the questionnaire and recruited participants. LL and JT analysed the data and wrote the manuscript. LL specifically focussed on global identity. JT specifically focussed on sufficiency orientation. PP and GR revised and edited the manuscript. All authors contributed to the article and approved the submitted version.

## Conflict of Interest

The authors declare that the research was conducted in the absence of any commercial or financial relationships that could be construed as a potential conflict of interest.

## Footnotes

1 We had additionally assessed multicultural experiences made in Germany based on Sparkman and Eidelman (2018). However, as it does not address our main research questions, we do not outline it here. Moreover, we had assessed a short 15-item version of the General Ecological Behaviour Scale (GEB) by Kaiser and Wilson (2000) with the main aim to give people the opportunity to express their pro-environmental engagement beyond mobility behaviour in order to reduce possible resentments. As a further addition to complement the flow of the questionnaire, we asked participants to estimate the relative CO_2_ emissions of airplanes, cars, and trains and gave the solutions, before assessing their willingness to compensate flying and use alternative train options. Analyses of these additional variables can be provided on request.

2 OSF Forum: https://bit.ly/3vbEGvh

3 As a robustness check for the results on flight-related CO_2_ emissions, we excluded *n* = 14 cases with values higher than two standard deviations above the median. In this reduced sample, we found neither correlations with global identity nor sufficiency orientation (see Supplementary Section “Supplement: Results”). Hence, these results should be treated with caution and may require future replication.

4 www.terran.eco

5 www.back-on-track.eu
