# Supplementary material for: Global Citizens – Global Jet Setters? The Relation Between Global Identity, Sufficiency Orientation, Travelling, and a Socio-Ecological Transformation of the Mobility System
Source: Front Psychol. 2021 Mar 30;12:622842. doi: 10.3389/fpsyg.2021.622842 (PMC8042256; doi:10.3389/fpsyg.2021.622842)
Supplement: Supplementary file 1 [file Data_Sheet_1.pdf]

Global Citizens – Global Jet Setters? The Relation Between Global Identity, Sufficiency Orientation, Travelling, and a Socio-Ecological Transformation of the Mobility System

Laura S. Loy, Josephine Tröger, Paula Prior, Gerhard Reese

Supplementary Material

1 Supplement: Methods

1.1 Power Analysis

We conducted two a priori power analyses, aiming at a test power of .80 at $\alpha = .05$. On the basis of prior research that reports relations between global identity, travel experiences, pro-environmental behaviour, policy support, and sufficiency orientation (Loy & Reese, 2019; Sparkman & Eidelman, 2018; Verfuerth et al., 2019), we determined a necessary sample size of $N = 270$ to detect correlations of at least $r = .15$. We expected a lower potential effect of our experimental manipulation to raise the salience of global identity in an online setting compared to prior experimental studies (Reese et al., 2015; Römpke et al., 2019). Thus, we determined a necessary sample size of $N = 204$ to detect small to medium group differences of at least $d = .35$.

In order to allow for a potential exclusion of participants after quality checks as well as potentially more complex analyses beyond our pre-defined hypotheses, we recruited an extended convenience sample of $N = 322$ participants.

1.2 Participant Characteristics

The recruited sample consisted of $N = 322$ participants (258 females, 61 males, 3 diverse; $M = 28.6$ years of age, $SD = 10.2$, range = 18-65). The sample was highly educated: university entrance certificate ($n = 140$, 43.5%), university degree or similar qualification ($n = 122$, 37.9%), PhD ($n = 20$, 6.2%), traineeship ($n = 6$, 1.9%), secondary school certificate ($n = 6$, 1.9%), pupils ($n = 2$, 0.6%), without any certificate ($n = 1$, 0.3%). The majority were students ($n = 171$, 53.1%), followed by employed people ($n = 113$, 35.1%), public officers ($n = 11$, 3.4%), unemployed ($n = 6$, 1.9%), and self-employed people ($n = 4$, 1.2%).

1.3 Exclusion of Outliers and Implausible Values

First, we examined the relative speed index and excluded $n = 2$ participants with values over 2.0 as speeders (Leiner, 2019). Second, we excluded $n = 3$ further participants who indicated that they did not speak German. All following analyses were conducted with a reduced data set of $N = 317$ participants.

Moreover, we examined plausibility and outliers for the variables with open answer formats. First, we examined the plausibility and completeness of *flight-related CO_2 emissions*. We excluded *n* = 15 cases whose answers were obviously not serious (e.g., indicated wrong or implausible destination names) or incomplete (indicated to fly but did not list any flight afterwards). In addition, we determined people with values more than two standard deviations (i.e., 50.53) above the median (i.e., 6.87), which was the case for *n* = 14 participants. As this number is rather large, we report results without their exclusion in the main manuscript and repeat the analyses with the reduced sample in Supplement 2 (see below).

Second, we examined the plausibility and completeness of the *willingness to accept a higher price of train alternatives*. We excluded *n* = 2 missing cases and *n* = 4 outliers. Outliers were determined as follows: We excluded two extreme cases (i.e., 100100 €). Then, we calculated the median (i.e., 100) and the standard deviation (i.e., 487.46), and defined people with values more than 2 SD above the median as outliers (i.e., 7007, 5050). Similarly, we examined the plausibility and completeness of the *willingness to accept a higher duration of train alternatives.* We excluded *n* = 2 missing cases and *n* = 4 outliers. Outliers were determined as follows: We excluded one extreme case (i.e., 1515100 h). Then, we calculated the median (i.e., 6) and the standard deviation (i.e., 62.32), and defined people with values more than 2 SD above the median as outliers (i.e., 801, 653, 400).

1.4 Measures

An overview on the psychometric properties of the measures is provided in Table 1.

1.4.1 Global Identity

Participants stated their agreement with five statements respectively on global self-definition (e.g., “I think of people all over the world as ‘we’”) and global self-investment (e.g., “I want to help people all over the world”) on a 7-point scale (*does not apply at all* to *fully applies*). The confirmatory factor analysis (CFA) of the two-dimensional model with correlating factors had a satisfactory model fit, $\chi^2(34) = 99.37$, $p < .001$; CFI = .97; TLI = .96; RMSEA = .078, 90% CI [.062, .094]; SRMR = .034. Factor loadings were between .76 and .89. The correlation of the two dimensions was .90. A one-dimensional model did not yield satisfactory model fit, $\chi^2(35) = 173.31$, $p < .001$; CFI = .93; TLI = .91; RMSEA = .112, 90% CI [.097, .126]; SRMR = .046. The two-dimensional model fit the data significantly better, $\chi^2_{diff}(1) = 111.07$, $p < .001$. However, the strong relation speaks for a second-order factor of global identity.

This is the full item list for global identity (German wording, see Loy & Reese, 2019; McFarland et al., 2012; Reese et al., 2015):

Answer scale: 1 = trifft überhaupt nicht zu, 2 = trifft überwiegend nicht zu, 3 = trifft eher nicht zu, 4 = trifft teilweise zu, 5 = trifft eher zu, 6 = trifft überwiegend zu, 7 = trifft voll und ganz zu

Global self-definition:

1. Ich fühle mich Menschen auf der ganzen Welt verbunden.
2. Ich denke an Menschen auf der ganzen Welt als „Wir“.
3. Ich habe viel mit Menschen auf der ganzen Welt gemeinsam.
4. Ich empfinde Menschen auf der ganzen Welt als eine Gemeinschaft.
5. Ich identifiziere mich mit Menschen auf der ganzen Welt.

Global self-investment:

6. Ich sorge mich um Menschen auf der ganzen Welt.
7. Ich fühle mich betroffen, wenn Menschen auf der ganzen Welt schlimme Dinge passieren.
8. Ich möchte ein verantwortungsvolles Mitglied der Weltgemeinschaft sein.
9. Ich fühle mich solidarisch mit Menschen auf der ganzen Welt.
10. Ich möchte Menschen auf der ganzen Welt helfen.

1.4.2 Travel Experiences

We asked participants how often in the past five years they had travelled in Europe on average per year on a 7-point scale (*never, less than one time, one time, two times, three times, four times, five times or more*), how long their respective longest stay had been (*in years, months, weeks*), how often in their lives they had travelled outside of Europe on a 7-point scale (*never*, *one time, two times*, *three times, four times, five times, six times or more*), and again, how long their respective longest stay had been (*in years, months, weeks*).

We used a measure by Islam and Hewstone (1993) to assess the quantity and quality of contact with people met during travelling with five items, respectively (see also Sparkman & Eidelman, 2018). Participants indicated on a 7-point scale (*never, very rarely, rarely, occasionally, often, very often, all the time*) how often they had met local people in a professional/university context, as neighbours, and as close friends, visited their homes, and engaged in informal conversations. A one-dimensional CFA had a satisfactory model fit, $\chi^2(5) = 14.67$, $p = .012$; CFI = .98; TLI = .96; RMSEA = .078, 90% CI [.037, .123]; SRMR = .027. Factor loadings were between .56 and .83. Moreover, they stated on 7-point semantic differentials to what extent they experienced the contact as *not at all equal* to *absolutely equal*, *very involuntary* to *very voluntary*, *very superficial* to *very intimate, not at all pleasant* to *very pleasant*, and *very competitive* to *very cooperative*. A one-dimensional CFA had a satisfactory model fit, $\chi^2(5) = 12.62$, $p = .027$; CFI = .97; TLI = .93; RMSEA = .070, 90% CI [.034, .106]; SRMR = .038. Factor loadings were between .54 and .78.

1.4.3 Calculation of Flight-Related CO_2 Emissions

We used the CO_2 calculator "KlimAktiv" (https://klimaktiv.co2-rechner.de/; Schächtele & Hertle, 2007, accessed May, 2020). In order to calculate the emissions of occasional flyers, we entered their indicated destinations. KlimAktiv then calculated the CO_2 emissions and we built a sum index per person. In order to calculate the emissions of frequent flyers, we entered the examples for the chosen reference categories (e.g., Hamburg – Munich for the distance category 500-1,000 km) and also built a sum index. Hence, the calculation for the frequent flyers is less precise than that for occasional flyers. However, we deemed it an unbearable burden to ask frequent flyers to list all of their flights in the last five years.

1.4.4 Refraining from Flight Travel

We asked participants how often in the past five years they had refrained from flying on a 7-point scale (*never, very rarely, rarely, occasionally, often, very often, always*). The original item in German wording read: Haben Sie in den letzten 5 Jahren bewusst auf Flugreisen verzichtet?

Answer scale: 1 = nie, 2 = sehr selten, 3 = selten, 4 = gelegentlich, 5 = oft, 6 = sehr oft, 7 = immer

We asked people who had refrained from flying in the last five years to indicate the reasons, with the option to select multiple answers. In the following, we give an overview on the frequencies:

- Climate protection (n = 184)
- Support of alternative ways to travel (e.g., train, n = 134)
- No desire or need to fly (n = 88)
- Flights too expensive (n = 54)
- Illness (e.g., travel sickness, flight anxiety, n = 29)
- Not able to pay for flights in general (n = 21)
- Private commitments (e.g., caring for family member, n = 18)
- Health-related reasons (e.g., cardiovascular disease, n = 8)
- Other reasons (open answer field; n = 22)

1.4.5 Flight Shame

Participants indicated their agreement to the statements “I feel ashamed/guilty that I have travelled by airplane” on 7-point scales (*does not apply at all* to *fully applies*). The items highly correlated (r_s = .76, p < .001) and we used mean scores for our analysis. The n = 26 participants who had not flown did not receive this question (missing values).

1.4.6 Policy Support

On a 7-point scale (*fully against* to *fully in favour*), participants rated five restrictive measures relating to cars (e.g., “creation of car-free city centres”), three restrictive measures relating to flying (e.g., “prohibition of private domestic flights below 1,000 km”), and three supportive measures relating to public transport and train travelling (e.g., “using public funds to provide free public transport”). We excluded one further assessed supportive item regarding biking from the final scale, because it reduced the model fit considerably. The CFA of the three-dimensional model with superordinate factor had an acceptable model fit except for CFI which is below the recommended threshold of .95 (Hair et al., 1998; Hu & Bentler, 1999), $\chi^2(41)$ = 98.20, p < .001; CFI = .93; TLI = .91; RMSEA = .066, 90% CI [.051, .082]; SRMR = .049. Factor loadings were between .42 and .78. Hence, the scale still needs improvement in future research, as also the average variance extracted (AVE) was below .50 (see Table 1).

In the following, we provide the full item list for policy support (German wording, see Loy & Reese, 2019; Tobler et al., 2012).

The original introduction sentence in German wording read: „Sind Sie grundsätzlich gegen oder für die folgenden politischen Maßnahmen, um CO_2-Emissionen zu reduzieren und so das Klima zu schützen? Bitte überlegen Sie dabei, welche Folgen diese Maßnahmen für Ihr Leben haben.“

Answer scale: 1= vollkommen dagegen, 2 = überwiegend dagegen, 3 = eher dagegen. 4 = unentschieden, 5 = eher dafür, 6 = überwiegend dafür, 7 = vollkommen dafür

Restrictive measures relating to cars:

1. CO_2-Bepreisung für Treibstoffe (Benzin, Diesel, Kerosin) *
2. Einführung eines generellen Tempolimits 130 km/h auf Autobahnen
3. Schaffung autofreier Innenstädte

4. Verbindliche Grenzen für die CO_2-Emissionen neuer Fahrzeuge
5. Keine Neuzulassung für Benzin- und Dieselfahrzeuge ab 2030

Restrictive measures relating to flying:

6. Verbot privater Inlandsflüge unter 1000 km
7. Verbindliches Kilometer-Budget für private Flugreisen pro Person
8. Verpflichtende Kompensationszahlungen für private Flugreisen **

Supportive measures relating to public transport:

9. Verwendung öffentlicher Gelder für den Ausbau eines klimafreundlichen Transportsystems (Busse, Bahnen)
10. Verwendung öffentlicher Gelder für die Einführung eines kostenlosen öffentlichen Nahverkehrs
11. Verwendung öffentlicher Gelder für Urlaubszuschüsse zu Bahn- oder Busreisen

Supportive measure relating to biking (excluded from final scale):

12. Verwendung öffentlicher Gelder für den Ausbau von Radwegen

* Infobox: Ein CO_2-Preis wird für jede Tonne Kohlenstoffdioxid (CO_2) gezahlt, die ausgestoßen wird. Er soll für Privatpersonen und Unternehmen einen Anreiz schaffen, weniger CO_2-Emissionen zu verursachen und klimafreundlicher zu wirtschaften. Der CO_2-Preis kann als CO_2-Steuer oder als CO_2-Emissionshandels-System umgesetzt werden.

** Infobox: Eine Reise mit einem Flugzeug verursacht CO_2 und trägt damit zur globalen Erwärmung bei. Durch eine Spende an Klimaschutzprojekte (sogenannte „Kompensationszahlungen“) sollen die entstandenen CO_2-Emissionen so weit wie möglich ausgeglichen werden. Klimaschutzprojekte setzen sich z. B. für den Erhalt von Wäldern ein oder fördern erneuerbare Energien. Kompensation kann das Klimaproblem nicht lösen, weil sie nichts an den eigentlichen CO_2-Quellen ändert. Sie ist eine vorübergehende Lösung für (noch) nicht vermeidbare Emissionen.

1.4.7 Sufficiency Orientation

We measured sufficiency orientation with six items from the sufficiency orientation short scale, capturing people’s attitude towards a low-carbon lifestyle (e.g., “I find it desirable to possess few things only”, Verfuerth et al., 2019) and added one further item from a former version (i.e. “I reject the idea that more and more is being consumed”, Henn, 2015). Moreover, we added seven items capturing people’s conviction that consumption reduction is a necessary means to environmental and climate protection (e.g., “I think renouncing consumption is helpful for environmental and climate protection”). Participants stated their agreement on a 7-point scale (*fully against* to *fully in favour*). We excluded two reverse-coded items because they built a separate method factor, leaving six positively formulated items for each dimension. We excluded the values of $n = 5$ cases (missing values). The CFA of the two-dimensional model with correlating factors had a satisfactory model fit, $\chi^2(53) = 109.14$, $p < .001$; CFI = .96; TLI = .95; RMSEA = .058, 90% CI [.045, .072]; SRMR = .047. Factor loadings were between .48 and .86. The correlation of the two dimensions was .73. As the AVE of the low-carbon lifestyle dimension was below .50 (see Table 1), the scale can still be improved in future research.

This is the full item list for sufficiency orientation (German wording; see Verfuerth et al., 2019):

Answer scale: 1 = stimme überhaupt nicht zu, 2 = stimme überwiegend nicht zu, 3 = stimme eher nicht zu, 4 = stimme teilweise zu, 5 = stimme eher zu, 6 = stimme überwiegend zu, 7 = stimme voll und ganz zu

Low-carbon lifestyle:

1. Durch meinen Lebensstil will ich möglichst wenige Ressourcen verbrauchen.[1]
2. Ich finde es erstrebenswert, wenig zu besitzen.
3. Ich finde es erstrebenswert, so viele Lebensmittel wie möglich selbst anzubauen oder herzustellen.
4. Ich halte all die neuen Dinge, die ständig verkauft werden, für eine große Ressourcenverschwendung.
5. Ich finde es überflüssig, dass es in unseren Supermärkten so eine riesige Auswahl an Produkten gibt.
6. Ich lehne es ab, dass immer mehr konsumiert wird.

Consumption impact:

7. Hoher Konsum führt zu ungerechten Verteilungsverhältnissen der natürlichen Ressourcen (z.B. Bodenschätze, Wasser) in der Welt.
8. Durch hohen Konsum steigt die Umweltbelastung.
9. Um Umweltbelastungen zu reduzieren, ist es auch notwendig den eigenen Konsum zu reduzieren.
10. Ich denke, Konsumverzicht ist hilfreich für Umwelt- und Klimaschutz.
11. Konsumverzicht reduziert das Ausmaß der Klimaerwärmung.
12. Ich bin davon überzeugt, dass wir mit einer Lebensweise, die den Ressourcenverbrauch maßgeblich reduziert, auch das Fortschreiten des Klimawandels verhindern können.

Excluded:

13. Mein Komfort ist mir wichtiger als eine sparsame Lebensweise. (umgepolt)
14. Mein Komfort ist mir wichtiger als eine Änderung meiner Lebensweise zu Gunsten eines höheren Umweltschutzes. (umgepolt)

Assessed but not used:

15. Ich bin ein genügsamer Mensch.

---

[1] We assessed a slightly shortened version recommended by Henn (2015) that gave no examples on particular resources people want to use less. The 2019 version was formulated as follows: „Through my lifestyle, I want to use as little resources as possible (e.g., water, energy, wood)“ (Verfuerth et al., 2019).

2 Supplement: Results

We repeated the correlation analyses involving flight-related CO_2 emissions excluding the $n = 14$ outliers determined above (see Supplement 1.3). We found neither significant correlations of flight-related CO_2 emissions with the dimensions of global identity, namely global self-definition ($r = .04$) and global self-investment ($r = -.00$), nor with the dimensions of sufficiency orientation, namely support of a low-carbon lifestyle ($r = -.10$) and the conviction that consumption reduction is helpful for environmental and climate protection ($r = -.07$, ps $\geq .098$). Hence, specifically our results involving flight-related CO_2 emissions should be replicated before drawing firmer conclusions.

3 Supplementary Tables

Table 1. *Descriptives and psychometric properties of the measures*

Variable	*n*	*M*	*SD*	range	items	α	ω	AVE
Global identity	317	5.31	1.19	1.30-7.00	10	.95	.95	.69
Self-definition	317	5.07	1.31	1.00-7.00	5	.91	.92	.69
Self-investment	317	5.55	1.18	1.00-7.00	5	.92	.92	.70
Frequency of travelling Europe	317	4.53	1.71	1-7	1	-	-	-
Duration of travelling Europe (in weeks)	317	20.26	104.26	0-1637	1	-	-	-
Frequency of travelling beyond Europe	317	4.49	2.29	1-7	1	-	-	-
Duration of travelling beyond Europe (in weeks)	317	16.50	49.59	0-728	1	-	-	-
Quantity of contact with locals	314	3.86	1.46	1.00-7.00	5	.84	.84	.51
Quality of contact with locals	314	5.59	0.91	2.00-7.00	5	.78	.78	.41
Flight-related CO_2 emissions (in tons per person)	302	23.05	50.35	0-590	1	-	-	-
Refraining from flight travel	317	3.54	1.86	1-7	1	-	-	-
Flight shame	291	3.12	1.68	1.00-7.00	2	-	-	-
Willingness CO_2 compensation	317	4.41	1.74	1-7	1	-	-	-
Amount CO_2 compensation (in €)	313	23.26	19.55	0-100	1	-	-	-
Accepted train price (in €)	311	102.52	88.63	1-1000	1	-	-	-
Accepted train duration (in h)	311	9.44	10.33	1-84	1	-	-	-
Policy support (3-dim)	317	4.88	1.11	1.08-7.00	11	.85	.88	.45
Sufficiency orientation	312	5.25	0.96	1.92-7.00	12	.90	.91	.50
Low-carbon lifestyle	312	4.82	1.08	1.00-7.00	6	.82	.82	.43
Consumption impact	312	5.69	1.05	1.83-7.00	6	.89	.90	.59

Note. α = Cronbach's alpha; ω = Raykov's omega; AVE = average variance extracted.

Table 2. *Bivariate correlations of the main study variables*

Variable	1	2	3	4	5	6	7	8	9	10	11	12	13	14	15	16	17
1. Global self-definition [a]																	
2. Global self-investment [a]	.94*																
3. Sufficiency orientation – low-carbon lifestyle [a]	.44*	.47*															
4. Sufficiency orientation – consumption impact [a]	.42*	.49*	.80*														
5. Frequency of travelling Europe [b]	.03	.03	-.14*	-.08													
6. Duration of travelling Europe	-.05	-.05	-.08	-.17*	.18*												
7. Frequency of travelling beyond Europe [b]	.08	.07	-.06	-.08	.17*	.16*											
8. Duration of travelling beyond Europe	.10	.10	.08	.04	.16*	.39*	.56*										
9. Quantity of contact with locals [a]	.24*	.21*	.09	.03	.20*	.16*	.28*	.25*									
10. Quality of contact with locals [a]	.27*	.27*	.08	.08	.11*	.04	.12*	.08	.37*								
11. Flight-related CO_2 emissions	-.08	-.12*	-.14*	-.15*	.31*	.00	.51*	.08	.22*	.21*							
12. Refraining from flight travel	.22*	.25*	.39*	.31*	-.02	-.04	-.17*	.02	.03	-.01	-.18*						
13. Flight shame	.35*	.40*	.46*	.45*	-.07	-.08	-.08	.03	.10	.05	-.04	.38*					
14. Willingness CO_2 compensation	.34*	.39*	.39*	.36*	-.05	.01	-.05	.08	.09	.12*	-.16*	.28*	.37*				
15. Amount CO_2 compensation	.21*	.22*	.20*	.17*	.01	-.01	-.01	-.00	.01	-.00	-.09	.20*	.22*	.27*			
16. Accepted train price	.15*	.16*	.22*	.19*	.07	.01	.05	.02	.13*	-.01	.00	.27*	.14*	.22*	.20*		
17. Accepted train travel duration	.13*	.12*	.17*	.17*	.00	-.03	-.06	-.01	.06	-.04	-.08	.15*	.11	.12*	-.02	.14*	
18. Policy support [a]	.43*	.48*	.65*	.65*	-.13*	-.12*	-.14*	.06	.04	.10	-.19*	.44*	.62*	.52*	.31*	.29*	.20*

Note. * $p < .05$. We used pairwise exclusion of missing cases. [a] Factor scores resulting from CFA were used. [b] Spearman correlations were calculated for ordinal variables; all others are Pearson correlations.

4 References

Hair, J. F., Black, W. C., Babin, B. J., & Anderson, R. E. (1998). *Multivariate data analysis* (4th ed.). Pearson Prentice Hall.

Henn, L. (December 2015). *Suffizienz – eine kleine Metaanalyse. Über Zuverlässigkeit, Vorhersagekraft und zusätzlichen Nutzen der Suffizienz-Skala*. Colloquium Personality and Social Psychology, Otto-von-Guericke University, Magdeburg, Germany.

Hu, L.-t., & Bentler, P. M. (1999). Cutoff criteria for fit indexes in covariance structure analysis: Conventional criteria versus new alternatives. *Structural Equation Modeling: A Multidisciplinary Journal*, *6*(1), 1–55. https://doi.org/10.1080/10705519909540118

Islam, M. R., & Hewstone, M. (1993). Dimensions of contact as predictors of intergroup anxiety, perceived out-group variability, and out-group attitude: An integrative model. *Personality and Social Psychology Bulletin*, *19*(6), 700–710. https://doi.org/10.1177/0146167293196005

Leiner, D. J. (2019). Too fast, too straight, too weird: Non-reactive indicators for meaningless data in Internet surveys. *Survey Research Methods*, *13*(3), 229–248. https://doi.org/10.18148/srm/2019.v13i3.7403

Loy, L. S., & Reese, G. (2019). Hype and hope? Mind-body practice predicts pro-environmental engagement through global identity. *Journal of Environmental Psychology*, *66*, 101340. https://doi.org/10.1016/j.jenvp.2019.101340

McFarland, S., Webb, M., & Brown, D. (2012). All humanity is my ingroup: A measure and studies of identification with all humanity. *Journal of Personality and Social Psychology*, *103*(5), 830–853. https://doi.org/10.1037/a0028724

Reese, G., Proch, J., & Finn, C. (2015). Identification with all humanity: The role of self-definition and self-investment. *European Journal of Social Psychology*, *45*(4), 426–440. https://doi.org/10.1002/ejsp.2102

Römpke, A.-K., Fritsche, I., & Reese, G. (2019). Get together, feel together, act together: International personal contact increases identification with humanity and global collective action. *Journal of Theoretical Social Psychology*, *3*(1), 35–48. https://doi.org/10.1002/jts5.34

Schächtele, K., & Hertle, H. (2007). *Die CO2 Bilanz des Bürgers: Recherche für ein internetbasiertes Tool zur Erstellung persönlicher CO2 Bilanzen* [The carbon footprint of the citizen. Investigation for an online tool to assess personal carbon footprints]. Umweltbundesamt. http://www.klimaktiv.de/media/docs/Studien/20642110_uba_die_co2-bilanz_des_buergers.pdf

Sparkman, D. J., & Eidelman, S. (2018). We are the "human family": Multicultural experiences predict less prejudice and greater concern for human rights through identification with humanity. *Social Psychology*, *49*(3), 135–153. https://doi.org/10.1027/1864-9335/a000337

Tobler, C., Visschers, V. H. M., & Siegrist, M. (2012). Addressing climate change: Determinants of consumers’ willingness to act and to support policy measures. *Journal of Environmental Psychology*, *32*(3), 197–207. https://doi.org/10.1016/j.jenvp.2012.02.001

Verfuerth, C., Henn, L., & Becker, S. (2019). Is it up to them? Individual leverages for sufficiency. *GAIA - Ecological Perspectives for Science and Society*, *28*(4), 374–380. https://doi.org/10.14512/gaia.28.4.9
